# The duration of untreated psychosis among U.S. Latinxs and social and clinical correlates

**DOI:** 10.3389/fpsyt.2023.1052454

**Published:** 2023-04-25

**Authors:** Maria M. Santos, Maya Kratzer, Jaqueline Zavala, Daisy Lopez, Jodie Ullman, Alex Kopelowicz, Steven Regeser Lopez

**Affiliations:** ^1^Department of Psychology, California State University, San Bernardino, San Bernardino, CA, United States; ^2^Department of Psychological and Brain Sciences, Boston University, Boston, MA, United States; ^3^School of Social Welfare, University of California, Berkeley, Berkeley, CA, United States; ^4^Department of Psychology, University of Miami, Miami, FL, United States; ^5^David Geffen School of Medicine, University of California, Los Angeles, Los Angeles, CA, United States; ^6^Department of Psychology, University of Southern California, Los Angeles, CA, United States

**Keywords:** duration of untreated psychosis, first episode psychosis, Latinx/Latino, English proficiency, immigration status

## Abstract

**Purpose:**

This study (a) documents the duration of untreated psychosis (DUP) and (b) examines both social and clinical correlates of DUP in a sample of U.S. Latinxs with first-episode psychosis (FEP).

**Methods:**

Data were collected for a longitudinal study evaluating a community education campaign to help primarily Spanish-speaking Latinxs recognize psychotic symptoms and reduce the DUP, or the delay to first prescribed antipsychotic medication after the onset of psychotic symptoms. Social and clinical variables were assessed at first treatment presentation. A sequential hierarchical regression was conducted using √DUP to identify independent predictors of the DUP. A structural equation model was used to explore the association between DUP predictors, DUP, and clinical and social correlates.

**Results:**

In a sample of 122 Latinxs with FEP, the median DUP was 39 weeks (*M* = 137.78, SD = 220.31; IQR = 160.39–5.57). For the full sample, being an immigrant and having self-reported relatively poor English-speaking proficiency and self-reported strong Spanish-speaking proficiency were related to a longer delay to first prescribed medication after psychosis onset. For the immigrant subgroup, being older at the time of migration was related to a longer delay. Self-reported English-speaking proficiency emerged as an independent predictor of the DUP. Although the DUP was not related to symptomatology, it was associated with poorer social functioning. Low self-reported English-speaking ability is associated with poorer social functioning *via* the DUP.

**Conclusion:**

Latinxs with limited English language skills are especially at high risk for experiencing prolonged delays to care and poor social functioning. Intervention efforts to reduce the delay in Latinx communities should pay particular attention to this subgroup.

## Introduction

The duration of untreated psychosis (DUP), or the delay between the onset of symptoms of psychosis and the initiation of adequate treatment (e.g., first prescribed antipsychotic medication) (1), is associated with poor short-term (2–4) and long-term outcomes for schizophrenia and related disorders (2, 5). The relationship emerges as early as first treatment presentation, with a longer DUP being associated with higher levels of positive symptoms and general psychopathology, and with lower quality of life (6).

U.S. Latinxs are at high risk for prolonged delays. Latinxs make up 18% of the U.S. population (7) and constitute the nation’s largest non-dominant racial/ethnic group. Over one-third of Latinxs are immigrants (8). A U.S. epidemiological household survey that focused primarily on anxiety, mood, and substance use disorders demonstrated that, in contrast to U.S.-born Latinxs, foreign-born Latinxs reported less use of past-year specialty mental health services and any service for mental health (9). Primary language was also found to be associated with mental health service use. Compared to those who reported speaking primarily English, those who reported speaking primarily Spanish reported less use of both specialty and any mental health services (9).

The U.S. epidemiological survey also showed disparities in mental health service utilization among immigrant Latinxs as a function of age at immigration. Use of any services and general medical services for mental health issues was lowest among those who immigrated to the U.S. between 13 and 34 compared to those who immigrated at or prior to age 12. It therefore may be useful to explore the relationship between age at immigration and the DUP as variability in immigrants’ patterns of mental health service use associated with age at immigration may influence delay to care after the onset of psychosis.

Research on influences on the DUP suggests that multiple factors determine the length of the delay to care after psychosis onset (10, 11). Consistent findings on the association between immigration status and the DUP (12–15) point to immigration status as a potential contributor of the delay to care after psychosis onset for populations which are heavily comprised immigrants, such as Latinxs. English language proficiency has been found to be robustly related to mental health service us by Latinxs in the U.S. (16). English language proficiency may therefore be another factor which leads to a prolonged delay to care for Latinxs with FEP.

Currently, there are initial reports of the DUP for Latinxs residing in the United States. The earliest data were obtained from a subsample of English proficient Latinxs derived from the RAISE Early Treatment Program (ETP) for FEP national study, which compared the effect of a comprehensive, multidisciplinary, team-based treatment approach for FEP (i.e., coordinated specialty care) for delivery in the U.S. community care system to usual care with a racially/ethnically heterogeneous sample (6). Specifically, 18 % (73 of 404) of the sample was comprised Latinxs from the states of California and Florida. The median DUP for the whole sample was 74 (*M* = 193.5; SD = 262.2) weeks, but limited DUP results for specific racial and ethnic groups were reported and no associations between DUP and race and ethnicity were observed. Data drawn from La CLAve, a communications campaign to reduce the DUP in a Latinx community in a Southern California catchment area, provided a more recent measure of the delay in this population. Participants were English and Spanish-speaking with FEP and their caregivers (17). In an initial examination of campaign outcome and language background on the DUP, they found that Spanish-speaking persons with FEP had longer delays to any treatment than English-speaking counterparts (Spanish speaking: *M* = 196.12, SD = 420.20; English speaking: *M* = 91.74, SD = 174.45) and longer delays to prescription of anti-psychotic medication (Spanish speaking: *M* = 293.84, SD = 476.65; English speaking: *M* = 111.10, SD = 179.57). Given that their main objective was to examine the education campaign’s outcomes on DUP, they did not examine social and clinical correlates of the DUP for their sample.

The current study builds on Lopez et al.’s (17) findings by investigating the DUP in greater depth by describing the characteristics of the DUP distribution, examining DUP correlates, and identifying determinants of the delay with the La CLAve sample. Specifically, the first aim of the current study was to document further the delay to first prescribed medication after the onset of psychosis with a sample of Latinxs with FEP. The second aim was to identify sociocultural correlates of the DUP for Latinxs with FEP with specific attention to immigration-related factors and language background. We tested the hypotheses that differences in the delay would be observed by immigration status and that self-reported English- and Spanish-speaking proficiency would be associated with the DUP. We expected that, among immigrants and those who report a lower ability to speak English and a greater ability to speak Spanish, a longer delay would be observed. Given the goal of identifying determinants of the DUP, we also aimed to identify the strongest predictor(s) of the DUP. We hypothesized that immigration status and English-speaking proficiency would predict the DUP, but Spanish-speaking proficiency would not predict it after accounting for the effect of immigration status and English-speaking proficiency. We also explored the relationship between age at immigration and the DUP. A third study aim was to examine the association between DUP and clinical and social functioning correlates at first treatment presentation. We tested the hypotheses that a longer DUP would be associated with higher symptom severity and poorer social functioning. We explored the association between the DUP and substance use. Our fourth aim was to explore the DUP as a mediator of identified DUP determinants and clinical and social functioning.

## Method

### Participants

Data were obtained (May 2014–December 2018) for a parent study (La CLAve) evaluating a community education campaign to help the Latinx community recognize psychotic symptoms and reduce the DUP (17). Patients were recruited from a public outpatient mental health center and a public medical center psychiatric emergency/inpatient unit. Eligible participants self-identified with a Latinx ethnic group. For this study, Latinxs are individuals who reside within the U.S. (18) and identify as Hispanic, Latin, Latino, Latina, Latinx, or with a specific ethnic group from Latin America [i.e., South America, Mexico, Central America, and the islands of the Caribbean whose inhabitants speak a Romance language (19)]. See Supplementary Table S1 for participants’ country of birth. Participants were ages 15–64, experienced a FEP, and met Diagnostic and Statistical Manual IV (DSM-IV) criteria for a schizophrenia-spectrum disorder or mood disorder with psychotic features. Ineligible participants met criteria for psychotic disorder due to a general medical condition or substance induced psychotic disorder or received prior treatment for psychosis (i.e., 1 year of continuous antipsychotic medication). Primary caregivers were also recruited. A total of 132 patient-caregiver dyads were enrolled. Twelve patients chose not to participate and permitted caregivers to serve as primary informant and 13 caregivers did not participate. All participants provided written informed consent or assent accompanied by parent/guardian consent. The University of Southern California’s Institutional Review Board approved study procedures.

### Assessments and materials

Patients and caregivers completed assessments separately in an outpatient clinic, inpatient unit, or their home based on their preferences and were enrolled in the study for follow-up assessment for 1 year. Study variable data are based on patient and caregiver report, as well as chart review and provider report when available.

#### Duration of untreated psychosis

The DUP multi-step assessment integrates the DSM-IV’s delineation of psychotic symptoms, Symptom Onset in Schizophrenia (SOS) Inventory frequency scale (20), Positive and Negative Syndrome Scale (PANSS) severity scale (21), and an assessment of type and initiation of treatment, including medication. DUP was defined as the number of weeks between onset of FEP and first prescription of antipsychotic medication (6), where onset of FEP was defined as the first week of psychotic symptoms corresponding to a score on the PANSS of 4 or more on positive subscale items 1, 3, 5, or 6 or on the general subscale item 9 (22). See Supplementary Figure S1 for a depiction of the process for determining the DUP for this study and for assessing the interrater reliability of DUP values.

Interrater reliability for DUP estimates was calculated using cumulative intra-class correlation coefficients (ICC) for rater pairs available for subsamples. The following are the ICC ranges for the DUP estimates: 0.87–1.00 (*n* = 45) for patient report; 0.66–1.00 (*n* = 50) for caregiver report; and 0.96–1.00 (*n* = 27) for cross-informant report. Test–retest reliability results at baseline and 6–8 week after baseline are *r* = 0.86 (*n* = 31) for patient report and *r* = 0.61 (*n* = 35) for caregiver report. Concurrent validity was obtained for our assessment of age of onset with that of the SOS; *r* = 0.88 (*n* = 23) for patient report and *r* = 0.99 (*n* = 27) for caregiver report.

#### Sociodemographic characteristics

The patient sociodemographic data included self-reports of gender (0 = male, 1 = female), age, date of birth, years in school, and employment status (0 = employed, 1 = unemployed). Language proficiency was based on self-reported ability to speak English and Spanish (4 = very well/muy bien; 3 = well/bien; 2 = poorly/mal; 1 = very poorly/muy mal) drawn from the linguistic proficiency scale of the Bidimensional Acculturation Scale for Hispanics (23). Immigration status was derived from patients’ self-reported country of birth (0 = Latin American country [immigrant], 1 = U.S. born). Additional immigrant data included: age at (the time of) migration, years in the U.S., and country of origin (0 = other Latin American country, 1 = Mexico).

#### Clinical characteristics

A clinical assessor with a Bachelor of Science in Nursing, over 20 years of experience conducting diagnostic and other clinician-based assessments on several externally funded studies, and who was trained to interrater reliability criterion levels for the Structured Clinical Interview for DSM-IV (SCID-I) for DSM-IV, administered the SCID-I to establish DSM-IV diagnoses. Diagnostic decisions were conducted in consultation with a study co-PI (A. Kopelowicz, M.D.) who has extensive clinical and research experience working with Latinxs with psychotic disorders, and the parent study PI (S. Lopez). The DSM-IV was also used to assess history of substance use and cannabis use rated on a 4-point (1 = no use, 2 = use, 3 = abuse, 4 = dependence) ordinal scale. The presence and severity of positive and negative symptoms were measured using the PANSS (21), which generated Positive Symptom, Negative Symptom, and General Psychopathology subscales. Level of social functioning was assessed using the Strauss–Carpenter Level of Function Scale (SC; functioning in the past year) (24), Social and Occupational Functioning Assessment Scale (SOFAS; highest level of functioning for at least a few months during the past year) (25), and Personal and Social Performance Scale (PSP; functioning in the past month) (26). SC has demonstrated effectiveness in predicting outcomes in schizophrenia (24) and high reliability and validity among a variety of populations (27–29). The SOFAS is a validated measure of problems in social, occupational, and interpersonal functioning and has exhibited reliability in the excellent range (30). The PSP has shown good validity and interrater reliability with inpatient psychiatric populations (26) and good test–retest reliability in a stable outpatient setting (31).

### Statistical analyses

A cut-off point of 6 months (long DUP: ≥24 weeks) was used to describe participants who were in a “critical period” during which it is particularly important to end the DUP. Although a “critical period” during which it is particularly important to end the DUP has not been definitively established (32), findings show that significant improvements in outcome can be achieved by reducing the DUP from 6 months to 1 month (32, 33). Differences in DUP and participant characteristics between immigration status groups (i.e., immigrants and U.S.-born Latinxs) were investigated using independent samples *t*-tests for continuous variables and Chi-square tests of independence for categorical variables. Bivariate correlations were used to examine associations between DUP and continuous language and immigration variables. A sequential multiple regression was then conducted to assess predictors of the DUP given the aim of identifying potential determinants of the DUP based on the research literature and this study’s correlational findings. Specifically, we examined whether English-speaking proficiency contributed significantly to the prediction of the DUP after accounting for the effect immigration status, and whether Spanish-speaking proficiency contributed significantly to the prediction of the DUP after accounting for the effect of English-speaking proficiency and immigration status. A structural equation model was estimated to explore the relationship between purported determinant(s) of the delay to care and the DUP (observed variables), and social functioning (latent variable). Goodness of fit of the model was assessed using χ^2^, CFI, and RMSEA. Statistical analyses were conducted with SPSS version 26 and EQS.

### Missing data and assumptions

Of the 132 dyads enrolled, 4 dropped out immediately after the screening and 3 at later assessment stages due to participant relocation, change in contact information, or decision to end participation. Two dyads dropped out because the patients could not participate due to illness severity. In addition, two patients, for whom illness onset was documented, never received an initial prescription of antipsychotic medication. Complete DUP data were therefore available for 121 dyads. Missing data were examined, and a review of separate variance *t*-tests suggested that there are predictors of missing data. For instance, the DUP variable was associated with systematic differences in other variables (i.e., PANSS score *p*s = 0.001–0.006; language proficiency scores *p*s = <0.001). We considered the data to be missing at random and missing data were imputed for 121 dyads with complete DUP data and the 2 dyads with available onset data using the Maximum Likelihood Expectation Maximization algorithm (34).

Variables were assessed to determine whether the assumption of normality was met. The DUP variable was positively skewed and was therefore normalized by square-root transformation (√DUP) for parametric tests. The DUP thereafter met assumptions of normality upon inspection of skew index scores which were less than the absolute value of three (highest was 1.626), and a kurtosis index less than the absolute value of eight (highest was 3.04), which is within normal limits (35). The DUP variable was also assessed for univariate outliers and one case was removed due to a *z*-score value of ≥ to the absolute value of 3.3, *p* > 0.001 (34). The participant’s DUP of 2209.43 weeks had a *z*-score value of 4.45. The final sample size of 122 did not include this participant, which appears to belong to a different population.

Untransformed and non-imputed data were used to describe the DUP for our sample of Latinxs (*n* = 120) and parametric tests were conducted with the imputed √DUP and other variables (*N* = 122).

## Results

### Descriptive statistics

Supplementary Table S1 presents descriptive statistics for the final sample of Latinxs with FEP and the US-born and immigrant subgroups with non-imputed data, as well as the count and percent missing data for each variable. Latinxs with FEP sampled were predominantly bilingual, male, in their mid-20s on average, unemployed, and diagnosed with a psychotic disorder, and reported a history of some use of substances including cannabis. Compared to their U.S.-born counterparts, Latinx immigrants were older and reported fewer years of education. Latinx immigrants with FEP reported Lower English proficiency and greater Spanish proficiency relative to U.S.-born counterparts. Immigrants reported less cannabis use compared to U.S.-born individuals. No differences were observed between the groups based on other variables presented in the table of descriptive statistics.

### DUP among Latinxs

**Figure 1:**
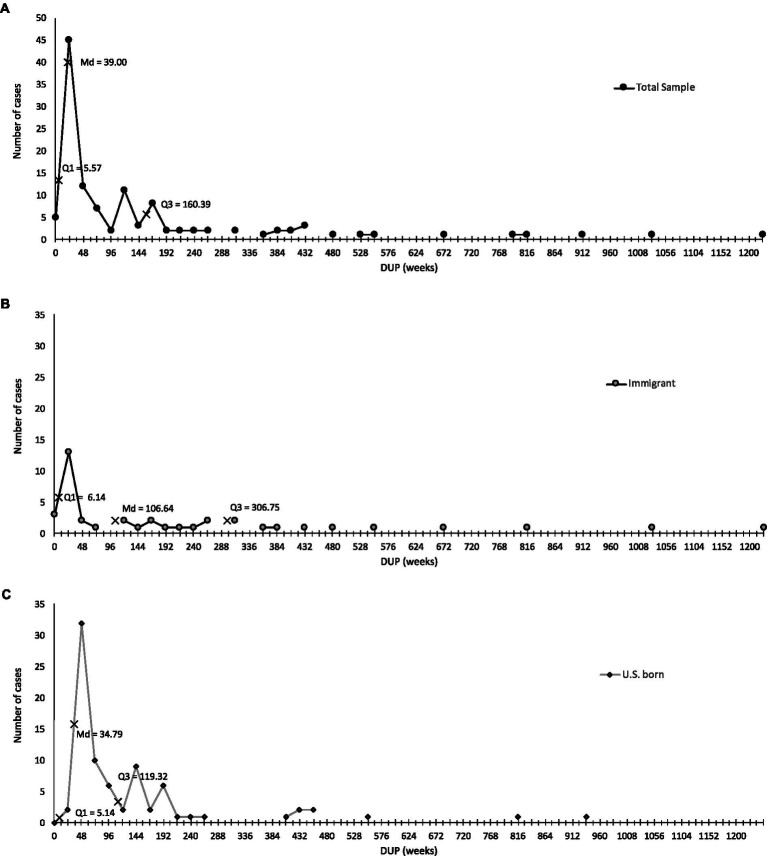
The delay to first prescribed antipsychotic medication after the onset of psychosis in a sample of U.S. Latinxs. **(A)** Distribution of DUP for full Latinx sample. **(B)** Distribution of DUP for immigrant Latinx sample. **(C)** Distribution of DUP for U.S-born Latinx sample.

The median DUP for the full sample was 39 weeks (*M* = 137.78, SD = 220.31; interquartile range [IQR] = 160.39–5.57). Figure 1A presents the distribution of the DUP for the full Latinx sample. At one end of the distribution, 25% of Latinxs had a DUP that was approximately 5.57 weeks or shorter. At the other end of the distribution, 25% of Latinxs had DUPs that were 160.39 weeks or longer. See Supplementary Table S1 for DUP weeks for the full sample, the two immigrant status subgroups, and across specific participant characteristics.

A cut-off point of 6 months was used to mark a ‘critical period’ during which it is particularly important to end the DUP (32, 33). In total, 41.67% (*n* = 50) of patients had a DUP which was <6 months and 58.33% (*n* = 70) patients were at or above this cut-off. Patients with a DUP < 6 months had a delay of just over 1 month (weeks: Md = 4.43; *M* = 7.3, SD = 7.68), whereas those with a DUP ≥ 6 months had a treatment delay of 34.3 months (weeks: Md = 149.14; *M* = 280.7, SD = 371.32).

### Correlates of the DUP among Latinxs

#### Sociodemographic variables

A significant difference in DUP weeks was observed between U.S.-born (√DUP = 7.65[6.55]) and immigrant Latinxs (√DUP = 10.97[9.35]), *t*(62.69) = 2.05, *p* = 0.04. As noted in Figures 1B,C, the distributions of U.S.-born and immigrants vary notably past the first quartile. Reports for U.S.-born Latinxs showed a median DUP of 34.79 (*M* = 100.84, SD = 164.08) weeks, whereas immigrant Latinxs experienced a median DUP of 106.64 (*M* = 211.65, SD = 291.84) weeks. Furthermore, 75% (60/80) of U.S.-born Latinxs had been prescribed initial medication by 119.32 weeks after psychosis onset, and DUP frequencies for immigrants showed that just 52.5% (21/40) of immigrants had also received a medication prescription by that time. Although just 75% (30/40) of immigrants were prescribed initial medication by 306.75 weeks after psychosis onset, a review of DUP frequencies for U.S.-born Latinxs show that 90% (72/80) of this subgroup had received an initial prescription by this point.

Table 1 presents the correlations between the √DUP and purported correlates. Lower English proficiency and greater Spanish proficiency were associated with a longer delay to first prescribed medication among Latinxs, whether immigrant or U.S.-born.

**Table 1:** Correlations between the DUP and sociodemographic and clinical and social functioning variables.

	Variables	1	2	3	4	5	6	7	8	9	10	11	12	13	14
1	√DUP	–													
2	English-speaking ability	−0.28**	–												
3	Spanish-speaking ability	0.18*	−0.12	–											
4	Age at migration	0.35*	−0.65***	0.37*	–										
5	Years lived in the U.S.	0.21	0.07	−0.2	−0.1	–									
6	Years in school	0.06	0.32***	−0.21*	−0.18	−0.16	–								
7	Positive symptoms	−0.06	0.15	−0.03	−0.25	0.05	0.16	–							
8	Negative symptoms	0.02	0.09	−0.03	−0.04	0.12	−0.02	−0.05	–						
9	General symptoms	0.01	−0.09	−0.16	0.09	0.06	0.004	0.27**	0.51***	–					
10	SC	−0.24**	−0.02	−0.11	0.04	−0.22	−0.004	−0.22*	−0.5***	−0.36***	–				
11	SOFAS	−0.26**	−0.02	−0.03	0.04	−0.23	−0.03	−0.18	−0.44***	−0.34***	0.92***	–			
12	PSP	−0.05	−0.06	0.04	0.08	0.03	−0.21*	−0.43***	−0.44***	−0.48***	0.37***	0.32***	–		
13	Substance use history	−0.19*	0.24**	−0.17	−0.28	−0.16	0.11	0.26**	−0.1	−0.05	−0.02	−0.03	−0.17	–	
14	Cannabis use history	−0.3***	0.38***	−0.28**	−0.45**	−0.08	0.25**	0.28**	−0.1	−0.04	0.02	0.05	−0.22*	0.78***	–

*p < 0.05, **p < 0.01, ***p < 0.001 (two-tailed). *N* = 122 for all variables, except age at migration and years lived in the U.S. for which *n* = 42. Analyses conducted with imputed data. Pearson’s r bivariate correlations conducted to examine all associations.

When considering only immigrant Latinxs, older age at the time of migration to the U.S. was associated with a longer DUP. Among immigrants, no differences in the DUP by Latin American country of birth (Mexico, other country) nor associations between the DUP and years lived in the U.S. were observed. For the full sample, no differences in the DUP by gender or employment status were observed, and no association with years in school was found. Small samples precluded an analysis of differences by diagnosis.

#### Clinical and social functioning variables

The √DUP was not statistically associated with positive, negative, or general psychopathology symptoms. However, a longer √DUP was associated with Strauss–Carpenter-rated poorer functioning in the past year and SOFAS-rated functioning during the three-month period of highest functioning in the past year. PSP-rated functioning was not associated with the √DUP. A longer delay to first prescribed medication after FEP onset was also associated with lower severity of substance use history and cannabis use history specifically.

### Determinants of the DUP

Results of a sequential multiple regression to identify predictors of the DUP are presented in Table 2. At Step 1, immigration status explained 4.2% of the variance in the delay to first prescribed antipsychotic medication, *F*(1,121) = 5.23, *p* = 0.02, with immigrants experiencing a longer DUP, *t*(130) = −2.29, *p* = 0.02. When English-speaking proficiency was entered at Step 2, the model accounted for an additional 4.6% of the variance in the DUP, *F*(2,121) = 5.71, *p* = 0.004, with English-speaking proficiency driving this effect. Lower self-reported English proficiency was related to a longer DUP, *t*(130) = −2.45, *p* = 0.02. However, at this step, immigration status did not remain a significant predictor of the DUP, *p* = 0.3. The model did not explain additional variance in the DUP when Spanish-speaking proficiency was entered at Step 3, *F*(3,130) = 4.45, *p* = 0.005. A review of coefficients confirmed that Spanish-speaking proficiency did not predict the DUP, *p* = 0.18 and that English-speaking proficiency remained the only significant predictor of the delay, *t*(130) = −2.57, *p* = 0.01.

**Table 2:** Sequential multiple regression of potential determinants of the delay to first prescribed medication after psychosis onset.

Variables	*B*	SE *B*	Beta	*p*	*R* ^2^	*R*∆	*p*
Model 1					0.04		0.02
Immigration status	−3.32	1.45	−0.2	0.02			
Model 2					0.09	0.05*	0.004
Immigration status	−1.64	1.58	−0.1	0.3			
English-speaking ability	−2.06	0.84	−0.24	0.02			
Model 3					0.1	0.01	0.005
Immigration status	−0.6	1.75	−0.04	0.73			
English-speaking ability	−2.16	0.84	−0.25	0.01			
Spanish-speaking ability	1.19	0.88	0.13	0.18			

Immigration status variable was dummy coded 0 = immigrant, 1 = U.S.-born. Language speaking ability was rated on a 4-point scale, where a higher score reflected greater self-reported ability to speak the language. *p < 0.05.

### Relationship between determinants, DUP, and clinical and social functioning

**Figure 2:**
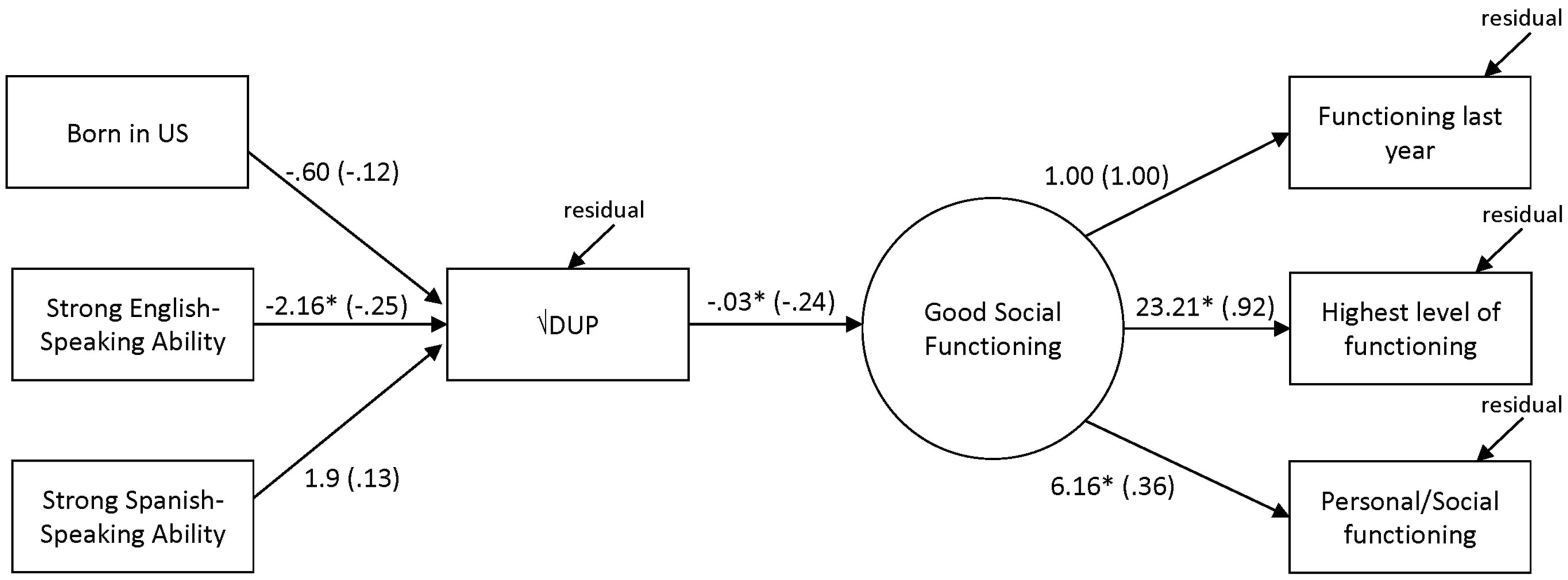
SEM of the relationships between determinants, DUP, and social functioning with unstandardized (standardized) coefficients. *p < .05. Rectangles represent observed variables and the circle the latent variable. Observed variables correspond to the following measures: self-reported immigration status (yes/no for born in U.S.), self-reported language proficiency (linguistic proficiency scale of the Bidimensional Acculturation Scale for Hispanics), functioning in the last year (Strauss-Carpenter Level of Function Scale; SC), highest level of functioning (Social and Occupational Functioning Assessment Scale; SOFAS), and personal/social functioning (Personal and Social Performance Scale; PSP).

Predictors in the SEM model were selected based on theoretical relevance. A total of seven measured variables were employed. The model was evaluated with the Chi-square statistic as well as the CFI and RMSEA. A CFI greater than 0.95 and a RMSEA equal of less than 0.05 are indicative of good fit (36). A structural equation model was estimated using maximum likelihood (ML) estimation to examine the relationships between observed purported determinant variables (immigration status, English and Spanish proficiency all measured variables) and the DUP (transformed, a measured variable), and a latent variable of social functioning with three scales serving each a measured variable serving as indicators (SC, SOFAs and PSP). There was evidence of excellent model fit, χ^2^(11, *N* = 122) = 14.12, *p* > 0.05, CFI = 0.99, RMSEA = 0.05. The model is present in Figure 2 with unstandardized and (standardized) coefficients included. The indirect effects of the demographic variables on social functioning were not significant. Squares represent measured variables and the latent variable is indicated as a circle. Lines denote direction of relations. Lines are also included to indicate residuals were estimated. Our path analysis to explore the relationship between English-speaking proficiency, the DUP, and clinical and social functioning revealed an indirect effect on functioning *via* the DUP.

## Discussion

We obtained support for our hypotheses on the association between factors that impact mental health service use among Latinxs and the DUP. Compared to their U.S.-born counterparts (median = 34.79), immigrant Latinxs (median = 106.64) reported longer delays to needed care after FEP onset. The disparity in the delay between the subgroups was elucidated in examining those with the longest DUPs. Although one-quarter of U.S.-born Latinxs had not received an initial prescription 2.29 years after psychosis onset, nearly half of immigrant Latinxs had not received an initial prescription by the same point in time. Latinxs with limited English proficiency and greater Spanish proficiency appeared to be at high risk for protracted delays to adequate care after a first psychosis episode as well. However, analyses showed partial support for our hypothesis regarding the relative contribution of these factors in explaining the DUP. Only English-speaking proficiency predicted the delay to adequate care after a FEP when accounting for the effect of immigration status and Spanish-speaking proficiency.

Our finding that Latinx immigrants experience a longer delay to care after FEP onset compared to U.S.-born Latinxs reflects immigrants’ service use patterns (37–39). Immigrants are generally less likely to use mental health services compared to their native-born counterparts. Health literacy is one factor which may lead to this pattern of service use and delays to timely care after psychosis onset and should be considered in the discussion of how to address this public health concern. Low health literacy acts as a barrier to immigrant service use (16, 40, 41) and may thereby contribute to longer DUPs among immigrants relative to natives (14). Latinx immigrants with FEP and their primary caregivers with low health literacy may not conclude psychosis is a problem that requires specialized treatment when making evaluations about the nature of the problem and indicated treatment. Findings obtained with a sample of Latinxs with FEP and their caregivers, many of whom were immigrants, showed that decreased psychosis knowledge and serious mental illness attributions are related to lower likelihood that patients and families will recommend seeking professional help for mental illness (41). In a subset of our sample, families sought care if they recognized the need for it (Hernandez et al., under review). (See Table 3 for two case examples that elucidate the role of the differential impact of health literacy on the delay to adequate care among immigrants and U.S.-born Latinxs.)

**Table 3:** Examples of the disparate impact of health literacy on the delay to adequate care by immigration status among U.S. Latinxs.

Immigration status	English-speaking ability	Spanish-speaking ability	Case description	DUP
Immigrant	Poorly	Very well	“Raul” was 42 years old at the time of first treatment presentation and is an immigrant from Mexico, who arrived in the U.S. at age 30, and whose primary caregiver was his wife of 18 years. His wife first observed changes 5 years before first treatment presentation, when he began to express jealously and concerns about his wife’s fidelity. A year later, he accused his wife of being unfaithful after he said he saw his wife in bed with another man. Concerned about her husband’s “celos” (jealously) and “inseguridad” (insecurity), his wife was not sure where to seek help and eventually sought help from her priest. However, it was not until sharing about her problems with various people in her social network that she considered the possibility that her husband was struggling with mental health issues. With the encouragement of her children’s social service providers and mental health counselors, she eventually helped her husband receive care at a local psychiatric emergency unit, thereby ending his delay.	311 weeks
U.S.-born	Well	Poorly	“Rafael” was 20 years old and living with his father, who was his primary caregiver, at the time of first treatment presentation. He had recently moved in with his father after his mother, who had raised him for most of his life, felt she could no longer handle her son as he had been “acting up” for some time. Within the week of moving in with his father, his father observed his son appear distracted when spoken to, as if he was trying to listen to others speaking even when no one else was present. He also caught his son talking to himself. The father immediately recognized his son’s behaviors as indications of mental illness. The father had observed these behaviors in his own mother, who had been diagnosed with schizophrenia. His father immediately sought mental health services after observing his son’s behavior and thus ended his son’s delay to care.	0 weeks

Families’ explanatory models of the problem that are based in culturally grounded conceptualizations of illness and differ from mental health provider models of illness can function as a barrier to mental health care access and extended delays to care for immigrants after the onset of illness (42) Rather than refer to psychosis as mental illness, Mexican-origin families have been found to utilize culturally meaningful illness categories, mostly notably *nervios* (nerves; (43)). *Nervios*, which is often used to refer to a wide range of distressing emotional and illness conditions, has been used by family members to label and understand serious mental illness including schizophrenia (44). Attributing the problem to *nervios* rather than mental illness may lead to families seeking help outside of the mental health care system in the immediate aftermath of illness onset and delay access to mental health care. For undocumented immigrant families, social policy may also lead to prolonged delays relative to other Latinx subgroups. In a study of Latino children and families, the percentage of children who needed and received services was much lower among children of undocumented parents compared to U.S. citizens and legal residents. Data suggested that stigma and fear of engaging with public agencies due to the vulnerable legal status of a caregiver deter caregiver help-seeking for children in need of care (45).

Our observations that Latinxs who report poor English proficiency and strong Spanish proficiency show extended delays to care converge with findings on Latinxs’ mental health service use. Limited English proficiency is robustly associated with underutilization of psychiatric services among U.S. Latinxs (16). Primarily Spanish-speaking Latinxs report less use of specialty and any mental health services compared to primarily English-speaking counterparts (9). Linguistic abilities appear to serve as a mechanism through which many Latinxs experience problems of access to mental health services and, in the case of those with FEP, extended delays to care after illness onset.

Although immigrants who arrive at older ages may be at lower risk of non-affective psychotic disorders (46), they may experience longer delays to care after the onset of psychotic disorder compared to those who arrive at a younger age. Our finding may be associated with differences in social context experienced by immigrants who arrive at younger versus older ages related to factors such as family separation and limited or nonexistent familial resources once in the U.S. Families play a crucial role in facilitating access to needed treatment after the onset of FEP (47, 48), particularly among immigrants (38). Families may help immigrants overcome obstacles to care, including low health literacy and inability to speak the dominant language in the host country (49, 50). Immigrants who arrive in the U.S. at younger ages may be more likely to arrive with family members and continue to benefit from this familial resource post migration, whereas those migrating at older ages may not.

### A Latinx subgroup at high risk for prolonged delays

Our study addresses the question about which factors—immigration status, English-speaking proficiency, Spanish-speaking proficiency, or some combination of these—predict the DUP. The findings showed that only English-speaking proficiency predicted the DUP above and beyond the effect of immigration status and Spanish-speaking proficiency. Latinxs with FEP with limited English proficiency are at greatest risk for long delays to care after FEP onset. Linguistic profiles observed among U.S. Latinxs (51, 52) and our sample suggest that Latinx immigrants are likely to be disproportionately affected.

The greater risk for a prolonged DUP for some Latinxs’ with FEP could be due to structural characteristics of the mental health system that facilitates access to services along the pathway to care for some segments of the population but not others. U.S. systems of care are set up to serve patients with good English-speaking proficiency as services are typically offered in English (50). Even in regions where efforts have been made to increase equity of mental health service access by meeting immigrant groups’ linguistic needs, such as California (50), individuals and families who do not speak the dominant language experience challenges in accessing needed care due to limited proficiency in the dominant language. An examination of the pathways to mental health care in a subset of the current study’s parent sample showed that even when some Latinxs with FEP and their families with poor ability to speak English did establish care, service systems were not equipped to offer care in Spanish, which extended delays to care (Hernandez et al., under review)^1^. The consequences of systems’ limited ability to meet the needs of linguistically diverse populations include hampered access to information on mental health care, service locations, appointment systems, and treatment affordability; psychiatric misdiagnosis (16) and greater delays to treatment for people with psychosis in particular (53). The health care system places many Latinxs with FEP and other populations without proficiency in the national language, and who are at greater risk for psychotic disorder in association with lack of language proficiency (46) at a disadvantage when the need for timely and effective care arises.

### The DUP and clinical and social functioning

The median delay to adequate care after the onset of a FEP in our sample of Latinxs from a heavily Latinx community in Southern California is 39 weeks. A longer DUP was observed in the RAISE community-based sample of individuals with FEP (Md = 74 weeks) (6). It is unclear whether the median DUP for our sample of Latinxs is comparable to that of the RAISE subsample of English proficient Latinxs since their median DUP was not reported. We identified a cohort of Latinxs with FEP at high risk for poor outcomes using a DUP cut-off point of 6 months (32, 33). Results showed that 58.33% of our sample were at increased risk of poor outcomes given a delay of 6 months or more.

Findings on the association between the DUP and prognostic variables (e.g., symptoms, functioning, and quality of life) at first treatment presentation have generally shown small or non-significant results, with statistically significant associations emerging at 6-and 12-month follow-up (3). Our examination of the relationship between DUP and symptoms at first treatment presentation yielded non-significant results. However, as expected, a longer DUP was associated with worse functioning in the past year and during the 3 months of worse functioning during the past year. In addition, the delay was longer among Latinxs with history of lower substance use and cannabis use in particular. Our findings on functioning and the DUP may suggest that the delay to a first prescription of antipsychotic medication contributes to worse illness by the initial treatment encounter. Alternatively, it may be that poorer patient functioning leads to a delay to treatment. With respect to the association between the DUP and cannabis use, it may be that the DUP contributes to reduced cannabis use and use of substances generally, perhaps because of poorer functioning. However, it seems more likely that use of substances precipitates service use by signaling the need for professional help to families who may not recognize the need for mental health treatment for psychotic illness.

Results of the path analysis indicated that lower ability to speak English results in a longer delay to treatment, which, in turn, may result in poorer functioning among Latinxs with FEP. Findings point toward the need to address the negative impact of limited English skills on accessing adequate care among Latinxs with FEP in the early stages of psychosis to prevent poor illness course.

### Implications for intervention and future directions

DUP reduction programs implemented with Latinxs with FEP should tailor early detection and intervention strategies to meet the needs of individuals with limited English proficiency. A reduction of the DUP was observed in. U.S. community in which an early detection campaign, “Mindmap,” was deployed within the catchment area of specialty first-episode service but not within a comparable control service’s catchment as part of a nonrandomized controlled trial. Individuals with FEP had to be able to communicate in English to participate in the study (54). A future iteration of the campaign’s detection strategies that target sources of delay to care should include mass and social media messaging, professional outreach, and rapid patient enrollment procedures conducted in Spanish and other languages spoken by non-English-speaking members of the target community. Lopez and colleagues developed an early intervention program to reduce the DUP which centered on a community education campaign to help the Latinx community, particularly Spanish-speaking residents, identify psychosis symptoms (55). Increasing psychosis literacy using a program delivered in Spanish represents an important step toward meeting the linguistic needs of Latinxs to achieve community-wide DUP reduction. More research is needed to better understand the reasons for which poor English proficiency contributes to delays in accessing needed care for psychosis. Results could inform the development of targeted interventions for reducing the delay for this Latinx group. Findings that the long delays are associated with poorer functioning even prior to treatment among Latinxs highlights the need to develop new or refine existing DUP reduction programs to effectively shorten the delay for Latinxs and mitigate the detrimental effects of the delay on illness and treatment course. This is underscored by findings that individuals with shorter DUPs experience the greatest clinical improvements after coordinated specialty care treatment (56). A next step to extend research on the DUP for U.S. Latinxs is to conduct a prospective study in which assessment of hypothesized DUP contributors precedes measurement of the DUP and measurement of the DUP precedes evaluation of individuals’ clinical and social functioning.

### Strengths and limitations

Eliminating mental health disparities that impact underserved, underrepresented, and hard-to-reach populations such as Latinxs requires documenting disparities and conducting research that can guide the service delivery system to better meet the needs of the population (57). This study meets these objectives by identifying a subset of Latinxs disproportionately affected by long DUPs and identifying factors that, if modified, may result in reduced disparities. Another strength of this study is that it disaggregated the sample by immigration status, which is essential for shedding light on differences in the delay in a heterogeneous group like Latinxs. A limitation of this study is that patients’ use of medication at the time of the initial assessment could have influenced psychopathology. Findings on the association between the DUP and psychopathology may therefore reflect the influence of medication use. Data did not permit an assessment of the role of medication on psychopathology. Mental health care sites from which this study’s sample was recruited did not offer early detection and intervention services for FEP. However, data used for this study were collected from members of a community within which a DUP reduction campaign was delivered. An evaluation of the campaign’s reach showed that the campaign message was received by the target community but did not result in a significant reduction in DUP (17). Given that the campaign did not result in a significant reduction in DUP, it is not likely that the campaign contributed to a significant underestimate of DUP for this sample.

## Conclusion

In the U.S., Latinxs with FEP with limited English language skills are especially at high risk for experiencing prolonged delays to care after psychosis onset and poor social functioning compared to counterparts with strong English language skills. Latinx immigrants who speak English poorly are likely to be disproportionately affected by this linguistic disadvantage and experience protracted delays. Efforts to reduce the DUP for Latinxs with FEP are most likely to succeed to the extent that they are designed to target mechanisms through which low English skills contribute to delays to adequate care.

## Data availability statement

The raw data supporting the conclusions of this article will be made available by the authors, without undue reservation.

## Ethics statement

This study was approved by the University of Southern California’s Institutional Review Board. Participants provided written informed consent or assent accompanied by the written informed consent of a parent/guardian.

## Author contributions

MS took the lead in conceptualizing and designing the study, conducting analyses and interpretating the data, and drafting and critically revising the manuscript. MK contributed to data management and analysis and preparation and revision of the manuscript. JZ and DL contributed to the methodology section of the manuscript. JU contributed to the data analytic plan, data analysis, and interpretation of results. AK made intellectual contributions to the design of the study and manuscript. SL made significant contributions to the design of the study and critical revision of the manuscript. All authors contributed to the article and approved the submitted version.

## Funding

The research leading to these results received funding from the National Institute of Mental Health under grant agreement nos 1R01MH103830 (PI: SL), 3R01MH103830-02SI (PI: SL), and K23MH119313 (PI: MS).

## Conflict of interest

The authors declare that the research was conducted in the absence of any commercial or financial relationships that could be construed as a potential conflict of interest.

## Publisher’s note

All claims expressed in this article are solely those of the authors and do not necessarily represent those of their affiliated organizations, or those of the publisher, the editors and the reviewers. Any product that may be evaluated in this article, or claim that may be made by its manufacturer, is not guaranteed or endorsed by the publisher.

## Footnotes

^1^Hernandez MY, Gamez D, Lee L, Mejia Y, Kalofonos I, Lopez SR. Visual pathways to care for Latinos experiencing first episode psychosis.
