# Supplementary material for: The duration of untreated psychosis among U.S. Latinxs and social and clinical correlates
Source: Front Psychiatry. 2023 Apr 25;14:1052454. doi: 10.3389/fpsyt.2023.1052454 (PMC10167038; doi:10.3389/fpsyt.2023.1052454)
Supplement: Supplementary file 1 [file Data_Sheet_1.docx]

Supplementary Material

The duration of untreated psychosis among U.S. Latinxs and social and clinical correlates

Maria M. Santos^1^*, Maya Kratzer^2^, Jaqueline Zavala^3^, Daisy Lopez^4^, Jodie Ullman^1^, Alex Kopelowicz^5^ and Steven Regeser Lopez^6^

*** Correspondence:** Maria M. Santos: [maria.santos@csusb.edu](mailto:maria.santos@csusb.edu)

| **Supplement Table 1** |  |  |  |  |  |  |  |  |  |  |  |  |
| --- | --- | --- | --- | --- | --- | --- | --- | --- | --- | --- | --- | --- |
| Participant Characteristics | | | | | | | | | | | | |
|  |  | U.S. Born | | Immigrant | | | |  | Full Sample | | | |
|  |  |  | |  | | | |  | N = 122 | | | |
|  |  |  |  |  |  | Missing | |  |  |  | Missing | |
| Characteristics |  | n | M(SD) or % | n | M(SD) or % | Count | % |  | n | M(SD) or % | Count | % |
| Immigration status (U.S. Born, Immigrant) |  | 80 | 65.57 | 42 | 34.43 |  |  |  | 122 | 100 | 0 | 0 |
| Gender |  |  |  |  |  |  |  |  |  |  | 0 | 0 |
| Male |  | 61 | 76.25 | 27 | 64.29 |  |  |  | 88 | 72.1 |  |  |
| Female |  | 19 | 23.75 | 15 | 35.71 |  |  |  | 34 | 27.9 |  |  |
| Age |  | 80 | 22.23(6.11) | 42 | 29.71(10.5)*** |  |  |  | 122 | 24.8(8.63) | 0 | 0 |
| Years in school |  | 66 | 11.95(1.54) | 39 | 10.05(3.73)** |  |  |  | 105 | 11.25(2.72) | 17 | 13.93 |
| Employed |  |  |  |  |  |  |  |  |  |  | 18 | 14.75 |
| No |  | 57 | 86.36 | 27 | 71.05 |  |  |  | 84 | 80.77 |  |  |
| Yes |  | 9 | 13.64 | 11 | 28.95 |  |  |  | 20 | 19.23 |  |  |
| Speaking ability |  |  |  |  |  |  |  |  |  |  |  |  |
| English |  | 66 | 3.62(.76) | 41 | 2.8(1.01) *** |  |  |  | 107 | 3.31(.95) | 15 | 12.3 |
| Spanish |  | 66 | 2.97(.98) | 40 | 3.75(.49)*** |  |  |  | 106 | 3.26(.91) | 16 | 13.11 |
| Age at migration |  |  |  | 41 | 14.91(8.51) | 1 | 2.38 |  |  |  |  |  |
| Years in U.S. |  |  |  | 41 | 15.97(7.21) | 1 | 2.38 |  |  |  |  |  |
| Country of birth |  |  |  |  |  | 2 | 4.35 |  |  |  |  |  |
| Mexico |  |  |  | 24 | 57.14 |  |  |  |  |  |  |  |
| Central America |  |  |  | 15 | 35.71 |  |  |  |  |  |  |  |
| South America |  |  |  | 3 | 7.14 |  |  |  |  |  |  |  |
| Diagnosis |  |  |  |  |  |  |  |  |  |  | 0 | 0 |
| Schizophrenia |  | 43 | 53.75 | 19 | 45.24 |  |  |  | 62 | 50.82 |  |  |
| Schizophreniform |  | 8 | 1 | 2 | 4.76 |  |  |  | 10 | 8.2 |  |  |
| Schizoaffective |  | 4 | 0.05 | 1 | 2.38 |  |  |  | 5 | 4.1 |  |  |
| Bipolar |  | 13 | 16.25 | 8 | 19.05 |  |  |  | 21 | 17.21 |  |  |
| Depressive |  | 3 | 3.75 | 2 | 4.76 |  |  |  | 5 | 4.1 |  |  |
| Psychosis NOS |  | 9 | 11.25 | 10 | 23.81 |  |  |  | 19 | 15.57 |  |  |
| Substance use history |  |  |  |  |  |  |  |  |  |  | 16 | 13.11 |
| No use |  | 9 | 13.43 | 12 | 30.77 |  |  |  | 21 | 19.81 |  |  |
| Use |  | 8 | 11.94 | 2 | 5.13 |  |  |  | 10 | 9.43 |  |  |
| Abuse |  | 8 | 11.94 | 4 | 10.26 |  |  |  | 12 | 11.32 |  |  |
| Dependence |  | 42 | 62.69 | 21 | 53.85 |  |  |  | 63 | 59.43 |  |  |
| Cannabis use history |  |  |  |  |  |  |  |  |  |  | 15 | 12.29 |
| No use |  | 12 | 17.39 | 16 | 42.11** |  |  |  | 28 | 26.17 |  |  |
| Use |  | 11 | 15.94 | 5 | 13.16 |  |  |  | 16 | 14.95 |  |  |
| Abuse |  | 13 | 18.84 | 11 | 28.95 |  |  |  | 24 | 22.43 |  |  |
| Dependence |  | 33 | 47.83 | 6 | 15.79 |  |  |  | 39 | 36.45 |  |  |
| PANSS |  |  |  |  |  |  |  |  |  |  |  |  |
| Positive symptoms |  | 78 | 28.64(6.07) | 41 | 27.41(6.88) |  |  |  | 119 | 28.22(6.36) | 3 | 2.46 |
| Negative symptoms |  | 75 | 20.03(7.57) | 41 | 18.24(8.22) |  |  |  | 116 | 19.4(7.82) | 6 | 4.92 |
| General symptoms |  | 76 | 39.24(10.73) | 41 | 38.39(10) |  |  |  | 117 | 38.94(10.44) | 5 | 4.1 |
| SC Total |  | 78 | 1.64(1.01) | 40 | 1.8(1.05) |  |  |  | 118 | 1.7(1.02) | 4 | 3.28 |
| SOFAS Total |  | 78 | 40.74(25.52) | 40 | 45.58(26.22) |  |  |  | 118 | 42.38(25.75) | 4 | 3.28 |
| PSP Total |  | 78 | 23.14(17.65) | 40 | 23.75(16.04) |  |  |  | 118 | 23.35(17.05) | 4 | 3.28 |
| DUP |  | 80 | 100.84(164.08) | 40 | 211.65(291.84) |  |  |  | 120 | 137.78(220.31) | 2 | 1.64 |
| *Note.* Non-imputed data are reported. Persons with FEP were asked how well they speak English and Spanish using Marin and Gomez’s (1996) rating scale: 4 = very well/muy bien; 3 = well/bien; 2 = poorly/mal; 1 = very poorly/muy mal. Substance use and cannabis use history were rated using an ordinal scale: 1 = No use; 2 = Use; 3 = Abuse; 4 = Dependence. PANSS=Positive and Negative Syndrome Scale subscales were rated on a 7-point scale (absent to extreme). SC=Strauss-Carpenter Level of Function Scale was rated on a 5-point scale (worst to best functioning) and the total score is the average of all subscale scores. SOFAS=Social and Occupational Functioning Assessment Scale was rated from 0-100. PSP=Personal and Social Performance Scale was rated from 0-100. DUP data reported are untransformed.  **p*<.05. ***p*<.01. ****p*<.001. | | | | | | | | | | | | |

**Supplement Figure 1**

The Delay to First Prescribed Antipsychotic Medication After the Onset of Psychosis in a Sample of U.S. Latinos


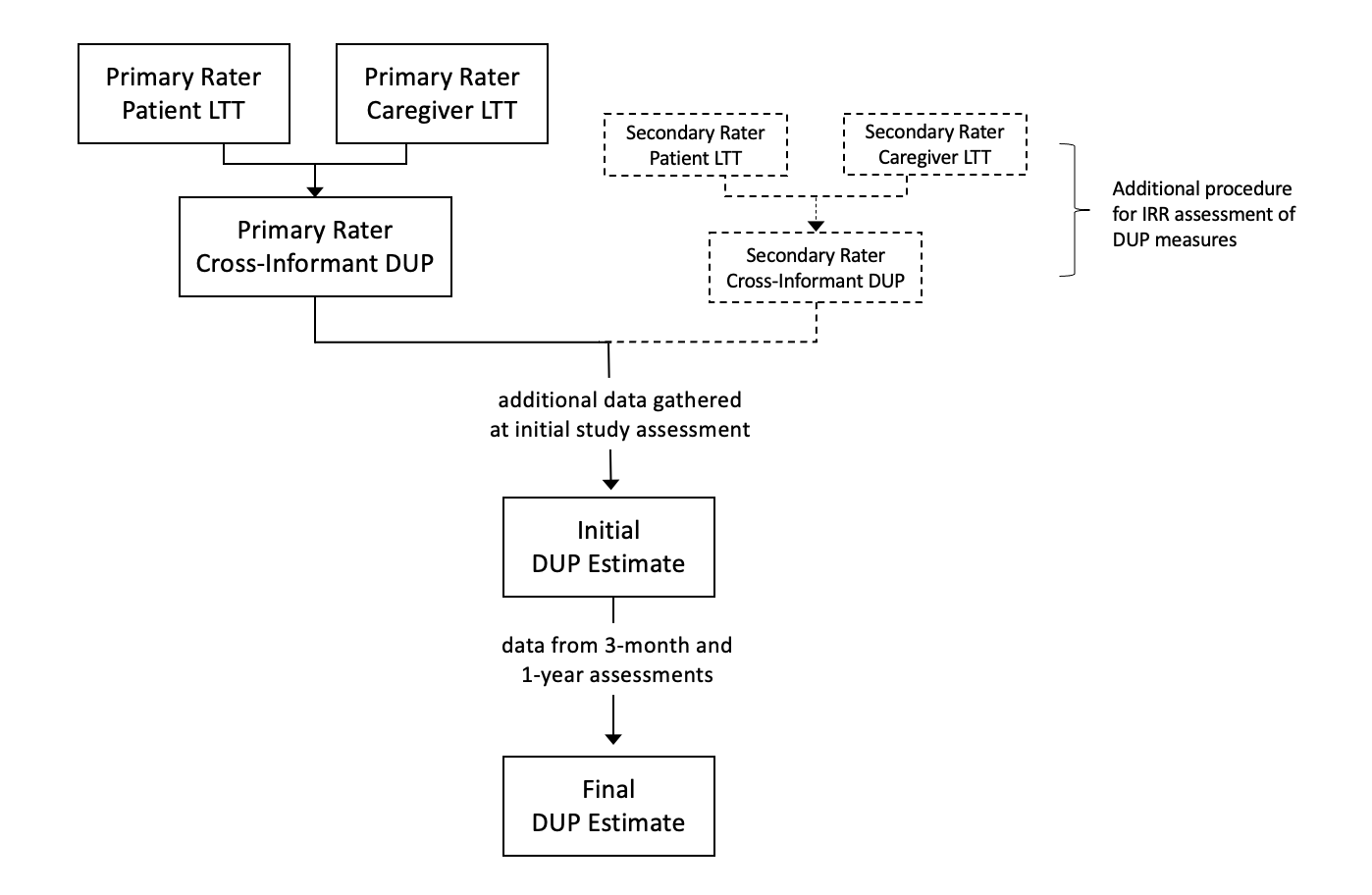


**Fig 1.** A process for determining the DUP was developed for this study. Using the LTT, a primary rater interviewed the patient and caregiver separately and then estimated a cross-informant DUP based on the patient and caregiver DUP reports. Project assistants later considered the cross-informant DUP and other initial assessment data, if warranted, to arrive at an initial DUP estimate. The initial DUP estimate was modified based on data acquired at follow up assessments, if needed, to arrive at a final DUP estimate. To assess LTT interrater reliability, the primary rater first interviewed the patient and caregiver while a secondary rater observed. Each rater independently estimated a cross-informant DUP based on the patient and caregiver DUP reports. Raters together addressed data discrepancies and considered other initial assessment data, if warranted, to arrive at a consensus of the initial DUP estimate. The initial DUP estimate was modified based on data acquired at follow up assessments, if needed, to arrive at a final DUP estimate.
